# SARS‐CoV‐2 rapid antigen test: Fast‐safe or dangerous? An analysis in the emergency department of an university hospital

**DOI:** 10.1002/jmv.27033

**Published:** 2021-05-09

**Authors:** Carola Holzner, Dirk Pabst, Olympia Evdoxia Anastasiou, Ulf Dittmer, Randi Katrin Manegold, Joachim Risse, David Fistera, Clemens Kill, Maximilian Falk

**Affiliations:** ^1^ Center for Emergency Medicine University Hospital Essen Essen Germany; ^2^ Institute for Virology University Hospital Essen Essen Germany

**Keywords:** inflammation, pandemics, SARS coronavirus

## Abstract

The use of Antigen point of care tests (AgPOCT) might be an essential tool to fight the coronavirus disease 2019 (COVID‐19) pandemic. Manufacturer information indicates a specificity of about 95% and there is a growing interest to use these tests area‐wide. Therefore, it is necessary to clarify whether AgPOCT can be used safely for “rule‐in” (detection of positive patients) and for “rule‐out” (valid negative testing). Two thousand three hundred and seventy‐five patients received polymerase chain reaction (PCR) testing and AgPOCT for severe acute respiratory syndrome coronavirus 2 (SARS‐CoV‐2) regardless of symptoms. The positive predictive value of symptomatic and asymptomatic patients was compared with a cut‐off threshold cycle (*C*_t_) value of ≤30 and in total. Five hundrded and fifty‐one patients tested positive for the SARS‐CoV‐2 virus by PCR, of whom 35.2% presented without symptoms. In all patients, regardless of their symptoms or *C*_t_ values, a sensitivity of 68.9% and a specificity of 99.6% were calculated for AgPOCT. In patients with *C*_t_ values ≤30, a sensitivity of 80.5% (95% confidence interval: ±1.62) and a specificity of 99.6% were shown for all tests (symptomatic/asymptomatic). Highly infectious patients (*C*_t_ ≤ 20), regardless of symptoms, were reliably detected by the AgPOCT. In infectious patients with *C*_t_ values ≤30, the test has a sensitivity of about 80% regardless of COVID‐19 typical symptoms, which is apparently less than the 96.52% specificity indicated by the manufacturer. Relevant improvement in test sensitivity by querying the patients who are symptomatic and asymptomatic is also not feasible. We strongly suggest that we critically question the use of AgPOCT for “rule‐out,” as they only provide a supposed safety.

## 1 INTRODUCTION

The severe acute respiratory syndrome coronavirus 2 (SARS‐CoV‐2) pandemic continues to obsess the world. Different strategies from “zero‐covid” with lockdown tightening up to a “life with the virus” are currently discussed. In the context of the so‐called “second wave” of the pandemic with the SARS‐CoV‐2 virus *(order Nidovirales, family Coronaviridae, genus Betacoronavirus, Species Severe acute respiratory syndrome‐related coronavirus)*, the question arises if rapid detection of infected persons, including asymptomatic ones, is possible.^1^, ^2^ In addition to the gold standard, the reverse transcription polymerase cahin reaction (RT‐PCR), a variety of antigen tests (so‐called “antigen point of care tests,” AgPOCT) are available.^3^ So far, these tests can be considered as an additive measure if standards are met.^4^, ^5^ The current literature shows weak documentation of the application of the test strategy in practice. Many validations are still in preprint^6^ or include smaller cohorts.^7^ There is an ongoing discussion for an antigen rapid‐test strategy to enable parts of “common” life, including, for example, tests at airports, nursing homes, and major events. In addition, hospitals are a central part of this discussion. Due to an increasing number of patients in the emergency room and scarce resources, both in terms of personnel and PCR testing, the question comes up if AgPOCT is an effective screening tool to initiate treatment of SARS‐CoV‐2 infection, accelerate de‐isolation of patients who do not have SARS‐CoV‐2 infection or prevent an outbreak in an inpatient setting. In hospitals and nursing homes, outbreaks not only pose organizational problems in a confined space but also pose serious health risks for a large proportion of patients and residents, as a more severe infection course is to be expected due to the risk profile. The aim of this study is to show whether AgPOCT is reliable and whether it could be used as the sole measure in the practice environment of a hospital and consecutively in public life not only for rule‐in (detection of positive patients), but also for rule‐out (valid negative testing).

## 2 MATERIALS AND METHODS

### 2.1 Design and participants

We retrospectively analyzed data from patients who presented for acute complaints in an emergency department of a German university hospital between October 16, 2020 and January 14, 2021. The general test strategy for detecting SARS‐CoV‐2 infection during this period involved testing each patient in the emergency department without and with SARS‐CoV‐2 symptoms (breathing difficulties, temp. >38°C and/or chills; cough, ansomy, ageusie, sore throat, fatigue, headache, limb pain, colds, gastrointestinal symptoms [nonspecific abdominal discomfort, diarrhea, vomiting]). Each patient received a nasopharynx smear according to the manufacturer's information for the performance of a PCR test and a simultaneous AgPOCT (Standard Q; Roche). We compared the positive and false negative tested patients based on their positive predictive value and set the cut‐off at a *C*_t_ value of 30. From the literature and the general approach, subjects with a *C*_t_ value below 30 are considered infectious. We focused on the correlation between CT value less than 30 and test results of AgPOCT and PCR testing.

### 2.2 Implementation

The RT‐PCR tests used were carried out on the basis of the manufacturers using Alinity in the SARS‐CoV‐2 assay (Abbott), Cepheid (GeneXpert), and RealStar SARS‐CoV‐2 RT‐PCR (Altona Diagnostics). The AgPOCT used was the standard Q (Roche). According to the manufacturer of the antigen test, a nasopharynx swab was performed over a nostril^7^ with repeated slight rotation after reaching resistance at the area of the posterior nasopharynx. The swab was then inserted into an extraction buffer tube where the liquid from the swab was extracted. Finally, the sample was applied to the test device and the result was read after 15–30 min. The collecting of the sample material for PCR testing was done in the same way via nasopharyngeal swab. Material collecting for both antigen and PCR testing was always carried out in patients by the same qualified nurse or physician.

### 2.3 Data collection

For this study, data from patients who received an AgPOCT in the emergency department were compared with PCR diagnostics performed in the emergency department and the Institute of Virology. In addition, the patient's history was used to ask about symptoms of SARS‐CoV‐2 infection. The patient history was evaluated according to symptoms (e.g., respiratory/thoracic, gastrointestinal, and general flu‐like symptoms, as well as additional symptoms, such as loss of smell and taste) and duration of symptoms. The data performed by the Institute of Virology included the type of testing, the manufacturer, and the determined *C*_t_ value. To achieve comparability of the *C*_t_ values of the different manufacturers, the testing was standardized using RealStar SARS‐CoV‐2 RT‐PCR according to the equations:

ct.Alinity=1701+(0.991×ct.Altona)

ct.cepheid=−0.227+(0.9990×ct.Altona)

### 2.4 Statistics

We created four‐field panels to calculate the specificities, sensitivities, and negative predictive values. For all continuous variables, the mean values ± standard deviation and the 95% confidence intervals were reported. Study prevalence was used to calculate negative predictive value. The calculations were performed using the scientific statistical modules scipy, pandas, and numpy of the programming language Python (version 3.0). The representations were created using the matplotlib module of the programming language Python.

### 2.5 Ethics

This study was approved by the ethics committee of the faculty of medicine of the University of Duisburg‐Essen on December 17, 2020. The study number is 20‐9769‐BO.

## 3 RESULTS

**Figure 1:**
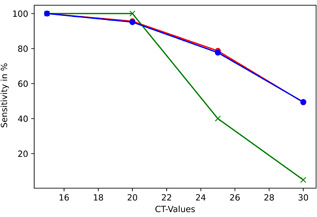
Course of sensitivities of rapid antigen testing in 551 SARS‐CoV‐2 positives. Blue‐curve: symptomatic; green‐curve: asymptomatic; red‐curve: combined. SARS‐CoV‐2, severe acute respiratory syndrome coronavirus 2

Out of 2375 patients, 551 (23,2%) tested positive for SARS‐CoV‐2 by PCR. Despite positive PCR, AgPOCT was negative in 172 patients. A deviation of a negative PCR test against a positive antigen test was shown in eight cases. The available data were used to calculate a specificity of 99.56% (95% confidence interval [CI]: 99.56 ± 0.26), a sensitivity of 68.87% (95% CI 68.87 ± 1.86), a positive predictive value (PPV) of 0.9793 (95% CI 0.9793 ± 0.0057) and a negative predictive value (NPV) of 0.9134 (95% CI 0.9134 + ‐ 0.011). This evaluation included all tests for all *C*_t_ values 1539 of the patients (64.79%) included in the study had typical coronavirus disease 2019 (COVID‐19) symptoms. For these symptomatic patients (*n* = 1539), the sensitivity of AgPOCT was 69.46% (95% CI: 69.46 ± 2.30) with an NPV of 0.8709 8 (95% CI: 0.8709 ± 0.0167) and the specificity was 99.51% (95% CI: 99.51 ± 0.34) with a PPV of 0.9858 (95% CI 0.9858 ± 0.0167). About one‐third of all patients (35.21%) included in the study did not show any COVID‐19‐like symptoms. In the AgPOCT testing of these asymptomatic patients (*n* = 836), sensitivity was 62.0% (95% CI: 62.0 ± 0.32) with an NPV of 0.9763 (95% CI: 0.9763 ± 0.0103) and specificity was 97.63% (95% CI: 97.63 ± 1.03) with a PPV of 0.9763 (95% CI: 0.9763 ± 0.0103). These results included all tests with all *C*_t_ values. To be able to make further differentiations regarding infectivity and statements of reliability of the AgPOCT, the positive antigen test results were compared to the *C*_t_ values of the PCR‐tests. Due to the deviations between PCR results and AgPOCT results, especially in the higher *C*_t_ values, sensitivities for the different intervals of the *C*_t_ values 0–20, 20–25, 25–30, >30 were determined separately. Up to a *C*_t_ value of 20, the sensitivity of the AgPOCT was 95.59% (95% CI: 95.59 ± 2.67) and the NPV 0.9868 (95% CI: ±0.0148), which is roughly the sensitivity reported in the manufacturer's information. For *C*_t_ values between 20 and 25, we found a sensitivity of 78.74% (95% CI: 78.74 ± 7.11) with a NPV of 0.9396 (95% CI: ±0.0414), for *C*_t_ values of 25–30 a sensitivity of 49.01% (95% CI: 49.01 ± 9.7) with a NPV of 0.8665 (95% CI: ±0.0660) and for *C*_t_ values greater than 30 a sensitivity of 12.63% (CI: 12.63 ± 6.68) and a NPV of 0.7911 (95% CI: ±0.0817). Figure 1 shows the course of the sensitivities.

To clarify the question of whether infection can be prevented with rapid antigen testing by filtering out infectious patients, we examined the case numbers for all tests below a CT value of 30. Regardless of whether the patients presented themselves with or without COVID‐19‐like symptoms, a sensitivity of 80.48% (95% CI: ±1.62) was found in direct comparison for all tests with a specificity of 99.56% (95% CI: ±0.271) and a PPV of 0.97 (95% CI: ±0.007) and an NPV of 0.95 (95% CI: ±0.0089) (Table 1).

**Table 1:** Four‐field table of PCR‐positive tested patients for *C*_t_ values ≤30

**AG‐test/PCR**	**PCR+**	**PCR−**	**Total**
Ag+	367	8	375
Ag−	89	1816	1905
	456	1824	2280

*Note:* Sensitivity: 80.48%; specificity: 99.56%; NPV: 0.95; PPV: 0.97.

Abbreviations: *C*_t_, threshold value; NPV, negative predictive value; PCR, polymerase chain reaction; PPV, positive predictive value.

The sensitivity for the symptomatic patients with *C*_t_ values ≤30 only (*n* = 423) was 79.67% (95% CI: ±2.06) with a specificity of 99.51% (95% CI: ±0.35), a PPV of 0.9853 (95% CI: ±0.0061), and an NPV of 0.9231 (95% CI: ±0.0136) (Table 2).

**Table 2:** Four‐field table of PCR‐positive tested symptomatic patients for *C*_t_ values ≤30

**AG‐test/PCR**	**PCR+**	**PCR−**	**Total**
Ag+	337	5	342
Ag−	86	1033	1119
	423	1038	1461

*Note:* Sensitivity: 79.66; specificity: 99.51%; NPV: 0.9231; PPV: 0.9853.

Abbreviations: *C*_t_, threshold value; NPV, negative predictive value; PCR, polymerase chain reaction; PPV, positive predictive value.

This sensitivity is just below the World Health Organization (WHO)‐required standard of 80%. Eight hundred and nineteen patients were asymptomatic. Of these patients, 30 had a positive PCR test with *C*_t_ values lower than 30. This corresponds to a sensitivity of 90.9% (95% CI: ±1.96%), specificity of 99.61% with an PPV of 0.9090 (95% CI: ±0.0196), and an NPV of 0.9961 (95% CI: ±0.0196).

## 4 DISCUSSION

The results of this show a lower sensitivity compared to the manufacturer's information (manufacturer: 96.52% [95% CI: 91.33–99.04] vs. 68.87% [95% CI: 68.87 ± 1.86]), with a higher number of subjects of a homogeneous patient population (manufacturer 426 vs. 2375) with lower confidence intervals.^8^ The sensitivity determined falls below the WHO standard of greater than 80% even after a distinction between symptomatic and asymptomatic patients ([95% CI: 69.46 ± 2.30] vs. [95% CI: 62.0 ± 0.32]).^9^, ^10^ However, this applies to all tested patients, regardless of their *C*_t_ values.

Assuming infectivity and thus the potential risk of transmission to others at *C*_t_ values below 30^11^, ^12^ it is worthwhile to take a closer look at the intervals and take them into account in the evaluation. The determined sensitivity results only for *C*_t_ values up to 20, which reliably is suitable to classify patients as negative or positive, regardless of whether the patients have symptoms or not. Below a *C*_t_ value of 20, patients are assumed to be maximally infectious.^13^ In this area, the AgPOCT does not reliably detect 10 out of 227 positive patients (sensitivity: 95.59% 95% CI: ±2.67). With a determined sensitivity of the AgPOCT of 78.74% (95% CI: 78.74 ± 7.11) from *C*_t_ values of 20–25, this rapid antigen test is below the WHO standard. Sufficient detection and therapeutic consequences for patients possibly being infectious due to SARS‐CoV‐2 cannot be made with the antigen testing result. One in five tests might be false negative. In this study in symptomatic patients with a *C*_t_ value of ≥25 the AgPOCT has only a sensitivity of 49.48% (95% CI: ±9.94). In asymptomatic patients, up to a *C*_t_ of 25, all patients showed a positive AgPOCT. 60% of all tests had false‐negative results at *C*_t_ values between 25 and 30. However, it should be noted that with *n* = 5 (*C*_t_: 25–30) the number of cases in this study is very small. For this *C*_t_ interval, the testing in our study is probably not representative because the majority of asymptomatic patients do not present themselves in a hospital and this screening took place within emergency patients who visited the hospital because of other complaints. Overall, the antigen test is able to detect positive patients with a sensitivity of 80.48% (95% CI: ±1.62) within patients who had a positive PCR with a *C*_t_ value of ≤30. Thus, one in five infectious patients is not recognized, with a specificity of 99.56%. A more representative examination outside a hospital should be considered to be able to make better statements about the test reliability of asymptomatic people, since like in this study, more symptomatic than asymptomatic patients are present in an emergency department. Within the hospital, the strategy has changed on the basis of this data to ensure that only patients with negative PCR are de‐isolated and therefore an antigen test is not sufficient. There must be no risk that one in five infectious patients reaches an inpatient setting undetected and thus an outbreak is risked.

In our opinion, the same applies in other areas of life, the regular “free‐testing” is currently being discussed, which should thus enable people to access nursing homes and other care facilities, airports, major events, etc. Due to the low sensitivity in asymptomatic people, large‐scale testing using this antigen rapid test method is certainly epidemiologically useful to recognize infected persons, but must not lead to a reduction of infection protection measures with a negative rapid test. In our view, this requires significant information measures for users and decision‐makers alike in order not to create new risks.

### 4.1 Limitations

This study retrospectively analyzes a collective of patients presented in a hospital. Therefore, the numbers of asymptomatic patients are not representative of the overall infections. Due to the sample collection by a large number of qualified staff, pre‐analytical factors must be considered. However, the same employee has taken the two tests compared and this setting reflects the real everyday situation of extensive tests, in which there are also no perfect laboratory conditions.

A second restriction is that too broad and loose periods of less than 7 and greater than 14 days were chosen for the documentation of the onset of symptoms. As a result, the analysis cannot relate to meaningful shorter periods of time and has been omitted

## 5 CONCLUSION

We conclude that the AgPOCT shows its strength in the reliable detection of highly infectious (*C*_t_ value to 20) patients, regardless of whether they have symptoms of SARS‐CoV‐2 infection or not. The higher the *C*_t_ value, detached from the symptoms, the less sensitive is the AgPOCT. An improvement in test sensitivity is also not relevant by distinguishing or querying the patients in symptomatic and asymptomatic. In both groups, similarly, low sensitivity is shown, although this is even lower among asymptomatic patients (95% CI: 62.0 ± 0.32) than in symptomatic patients. In all infectious patients regardless of COVID‐19‐like symptoms, defined as patients with a *C*_t_‐value of ≤30, the AgPOCT achieves a sensitivity of 79.7% and therefore is clearly lower than the sensitivity reported by the manufacturer. The results of this study may be transferred to AgPOCT‐Kits of other manufacturers as the test targets and the reported sensitivities are similar.^3^, ^14^ For a final conclusion further studies with different AgPOCT‐Kits need to be carried out. Potentially, PCR testing might not be necessary with a positive AgPOCT. The tests for the detection of highly infectious patients up to a *C*_t_ of 20 are particularly suitable. However, it is not possible to exclude infection or an infectious SARS‐CoV‐2 carrier. According to our study, the application of the test in, for example, hospitals, nursing homes, schools or after returning from high risk areas is therefore suitable for the detection of individuals, which would thus be detected as infectious and could be prevented from spreading by positive testing, but not for categorical negative testing. Due to our study results, we strongly suggest that the use of the antigen rapid tests for “rule‐out” should be critically questioned, as they suggest a supposed safety that cannot be scientifically supported. Nevertheless, this test procedure enables the rapid and easy detection of approximately two‐thirds of infectious patients in the context of large‐scale screening, even without or with only minor nonspecific symptoms, which otherwise go undetected. However, this enormous epidemiological advantage must be supplemented by an informed and responsible handling of negative test results.

## CONFLICT OF INTERESTS

The authors declare that there are no conflict of interests.

## AUTHOR CONTRIBUTIONS

Carola Holzner, Dirk Falk, and Maximilian Pabst contributed to statistic analysis, data interpretation, writing. Olympia Anastasiou, David Fistera, Randi Manegold, and Joachim Risse contributed to data acquisition. Clemens Kill and Ulf Dittmer contributed to proof‐reading and supervision.

### PEER REVIEW

The peer review history for this article is available at https://publons.com/publon/10.1002/jmv.27033

## Data Availability

The data that support the findings of this study are available on request from the corresponding author, (Dr. med. Carola Holzner). The data are not publicly available due to patient privacy (European general data protection regulation).
